# Thoughts, Past and Present

**Published:** 1894-12

**Authors:** 


### Editorial.

### THOUGHTS, PAST AND PRESENT.

The closing month of the year naturally tends to reflection, and the usually careless mind, as the days draw to the end, steps aside from the turmoil of daily life to consider the relations of the past with the present, the future being largely left for pleasant dreams.

With this idea impressing us, we give to our readers some of the thoughts that have forced themselves into prominence during the past year in connection with dental journalism and professional duty. Our own journal has claimed so much of our care that a short *résumé* of its history may serve to revive pleasant memories and perhaps bind more closely the interest of those who have followed its fortunes through the earlier years of its active life.

The Journal, under the name of *The Independent Practitioner*, was established by the original syndicate in New York, with the idea, as understood, that the dental profession might have a medium for an independent expression of thought upon all subjects connected with its welfare. While this was an important feature, it was also hoped to eliminate influences of a non-professional character from it, and aid in educating dentists to a higher standard of thought and action. That this was accomplished to a large degree by the controlling management is now a matter of history.

That this influence should have a wider field and a more enlarged opportunity was felt by many, and to effect this a company was formed to purchase *The Independent Practitioner* and give it the best conditions possible, to still further elaborate the good qualities of the journal named. This was finally accomplished, and the stock was rapidly absorbed, principally in the Eastern States.

The name of the journal was changed to that now held, and it has occupied a prominent position among the dental periodicals for the past six and a half years, and for four and a half years under the present editorial management.

During this period the Journal has steadily made its way as an exponent of a higher intellectual culture and a broader professional standard. The company very liberally gave the editor freedom to act as his judgment might dictate within certain limits, and these were in entire accord with his own conception of duty.

The work entailed great labor upon a few earnest men. They, through all the years it has existed, have devoted time and money to effect its permanent establishment, with no other reward in prospect but that dentists might have at least one journal not complicated with outside influence foreign to its best welfare. As the years passed on this labor was increased and the responsibilities heavy, but these have been cheerfully borne in the interest of the profession.

The apathy so generally manifested is a reproach to the dentistry of the United States. No profession can have a healthy life under its malign influence, and, unless a change is brought about, dentistry in America must abandon its egotistic assumptions or eventually be rated below the professional standing of that existing among all civilized peoples. A profession cannot exist without an advanced professional spirit, and without this it has no claims to recognition.

What do we mean by professional spirit? That feeling which regards the advance of the calling superior to all selfish considerations. With this definition how do we stand? We have, first, a large proportion who never subscribe for a journal. They may be rated as unworthy of any consideration whatever, and yet they are the dead weight, the nightmare, of our professional life. Secondly, the men who take a cheap journal made up of excerpts from original thinkers, and care for these periodicals only as they contain novelties on their advertising pages. Thirdly, a class who read, but will do nothing to aid by contributions or personal influence. Fourthly, a class who will at any time barter the products of their societies for money, and are ever ready to "crook the pregnant hinges of the knee" to capital. Their idea of professional excellence does not rise above the sordid principle of trade. Fifthly, men who are imbued with all the attributes of good professional men, but are negative in character, and ever willing to have the work of the profession transferred from their own to other shoulders. This, in brief, is the analysis of character met with among the antagonizing elements that bar the way and have been a dead weight upon piofessional progress.

With the fourth class we desire to have a word. There are several examples where it has bartered its work for a consideration. We hold that when the American Dental Association parted with the publication of its proceedings, it sacrificed its place of honor and dampened its professional spirit, and it rests under the incubus of having sold its birthright. The example has been baneful to itself and to others. We maintain that every scientific society, to be true to itself and regardful of its honor, must make the sacrifice to print its own proceedings and that by its own efforts. If it cannot do this, there is little reason for its existence. If it transfers its own work to others by the method indicated, it seals its own doom.

The example it set has been followed by many associations, and we have been treated to the shameful bartering of products all over the country, and giving these to the highest bidder with as much alacrity and disregard of ethical principles as the auctioneer sells his goods. That this is a disgraceful condition of affairs there can be no question, and the so-called profession that permits it in the code is surely bound to retrograde. A profession, to be such, must be above reproach. Better far that societies should perish than that this should continue.

The bright part of this otherwise dark picture is that some societies, and we could name them, have held the standard of professional life so high that no temptations, however golden, could reach them. These are the saving elements of the profession, and will, we are sure, lead it out of the dismal swamp of an isolated and selfish existence.

Few, we think, appreciate the full value of a journal such as this. It cannot come in competition with any other journal, for its aims are not in any sense of a selfish character. It has in the past endeavored to be an educating medium, to lead and not to follow, to impress on its pages a high standard of excellence in matter and in the quality of its work. The trivialities of journalism, as it is understood elsewhere, can have no place on its pages. It has endeavored to teach by example and precept that a profession to be worth the naming must be dignified. That "cap and bells" may do for the amusement of the unthinking crowd, but not for those who accept higher things. Its aim has been to lead the colleges of this country to as high a standard in professional education as may be consistent with existing circumstances. It holds the individual as the type of the mass, and as the separate parts are improved will the body become perfect. Hence we demand that each man who enters dentistry shall be taught, not only to honor it, but be forced to obey its highest precepts. In a word, the JOURNAL stands for the best that is obtainable in education, in ethics, and in general culture.

This journal was established and will be continued upon the basis as originally planned. There will be no compromise with the evils named. It hopes to be able to meet the best thought of the profession and to advance with it. It will demand the highest standard attainable in college, professional, and individual work. With these aspirations we close the year and welcome the next, confident that, whether we are sustained as fully as we should be or not, sooner or later dentistry will awake to the deep responsibility of its position.

The present is a fruitful period for good resolutions, the past we cannot change, but if it has had its proper value it has moulded thought and laid the foundations for a more satisfactory future.

The period demands a close introspection in regard to our ethical and secular duties, and these cannot be too closely scanned. It is the lack of this that gives the dental profession its shifting and heedless elements.

The duty of the hour seems to us to be to sustain unswervingly the highest manifestations in the profession, loyalty to it is the first consideration, and then will follow that other and correlated virtue, a readiness to make sacrifices for its advancement.

Progress is always slow. The years come and the years go, and the active worker therein fails to see much change, but the tide is ever sweeping onward and the grains of sand are ever becoming more rounded, ready to polish the angular surfaces of succeeding particles. This is the mission of humanity, as we understand it, and to meet this and prove an effective agent journals are necessary.

The journal of the present must be in advance of that of the past. It must be true to the enlarged standard of human thought and sustain the faltering and educate the ignorant. This has been the mission of the International Dental Journal, and it should receive the undivided support of all who make dentistry an ever-growing profession.

With this aim and these hopes we permit the year 1894 to pass into history without regret, and welcome the new birth with renewed aspirations for a higher development and enlarged opportunity.

